# Factors associated with professional identity of nursing interns in China: a systematic review and meta-analysis

**DOI:** 10.1186/s12909-026-09692-9

**Published:** 2026-06-19

**Authors:** Yao Yang, Ying Li, Yan Qiu, Ruwen Zheng, Jun Qian, Weifen Qiu

**Affiliations:** 1 Dali University, Dali, Yunnan China; 2 Anning First People’s Hospital Affiliated to Kunming University of Science and Technology, Kunming, Yunnan China; 3 Anning Traditional Chinese Medicine Hospital, Kunming, Yunnan China

**Keywords:** Nursing interns, Professional identity, Associated factors, Meta-analysis, China

## Abstract

**Background:**

This study aimed to identify factors associated with professional identity among nursing interns in China. Such evidence may help inform educational and clinical mentoring strategies to cultivate and strengthen professional identity during clinical internship, and may contribute to future retention among nursing interns, thereby supporting nursing workforce stability and improving the quality of nursing care.

**Methods:**

CNKI, Wanfang, VIP, the Chinese Biomedical Literature Database, PubMed, Web of Science, Embase, CINAHL and the Cochrane Library were searched from database inception to 20 January 2026. Cross-sectional studies involving nursing interns in China and reporting professional identity and its associated factors were included. Methodological quality was assessed using the quality assessment criteria for cross-sectional studies recommended by the Agency for Healthcare Research and Quality. Meta-analysis was performed using RevMan 5.4, with the correlation coefficient r used as the effect size.

**Results:**

A total of 48 studies were included, comprising 41 Chinese-language and seven English-language publications. Overall, 42 factors associated with professional identity were identified; 12 were included in the meta-analysis and 30 were synthesised descriptively. The meta-analysis showed that humanistic caring ability (*r* = 0.77, 95% CI: 0.48–0.91), medical narrative competence (*r* = 0.64, 95% CI: 0.60–0.69), professional self-efficacy (*r* = 0.62, 95% CI: 0.49–0.72), perceived professional benefit (*r* = 0.62, 95% CI: 0.57–0.66), clinical learning environment (*r* = 0.53, 95% CI: 0.18–0.77), psychological resilience (*r* = 0.52, 95% CI: 0.39–0.62), social support (*r* = 0.52, 95% CI: 0.34–0.66), perception of hospital caring climate (*r* = 0.45, 95% CI: 0.31–0.57), and perceived teacher caring (*r* = 0.36, 95% CI: 0.22–0.50) were positively associated with professional identity. Role stress (*r* = -0.32, 95% CI: -0.38 to -0.25) and anxiety (*r* = -0.14, 95% CI: -0.19 to -0.10) were negatively associated with professional identity. The association between clinical practice behaviour and professional identity was not statistically significant (*r* = 0.41, 95% CI: -0.03 to 0.73).

**Conclusion:**

This study showed that professional identity among nursing interns in China was associated with multiple factors. Humanistic caring ability, clinical learning environment, psychological resilience, professional self-efficacy, perceived teacher caring, hospital caring climate, medical narrative competence, perceived professional benefit and social support were associated with higher professional identity, whereas role stress and anxiety were associated with lower professional identity. The association between clinical practice behaviour and professional identity remained inconclusive. Nursing colleges and internship hospitals should support the development of professional identity among nursing interns by optimising the clinical learning environment, strengthening clinical mentoring support, enhancing psychological resources and reducing negative emotional experiences.

**Supplementary Information:**

The online version contains supplementary material available at 10.1186/s12909-026-09692-9.

## Background

Professional identity refers to a professional self-concept shaped by occupational characteristics, professional values and professional beliefs, and is an important outcome of professional socialisation [1]. For nursing interns, clinical internship represents a critical transition from the student role to the future nurse role. During this period, interns translate theoretical knowledge into clinical practice, learn professional norms, understand nursing responsibilities, experience nurse–patient relationships and develop perceptions of nursing values and role identity in real clinical settings. Therefore, clinical internship is a key stage in the formation and development of professional identity among nursing interns [2].

Professional identity is important for nursing education and workforce development. Previous studies have shown that it is closely associated with professional engagement, professional commitment, job satisfaction and retention intention [3–6]. Nursing interns with stronger professional identity may be more likely to recognise the value of nursing, develop positive clinical learning experiences and demonstrate stronger professional responsibility and willingness to remain in nursing. Conversely, lower professional identity may be associated with reduced professional confidence, greater difficulty in role adaptation and increased risk of leaving the nursing profession. Globally, nursing workforce shortages remain a major challenge for health systems. The State of the World’s Nursing Report 2025 reported a global shortage of approximately 5.8 million nurses [7]. In China, although the number of registered nurses reached 6.062 million by the end of 2025, with 4.32 registered nurses per 1,000 population [8], nursing workforce training and retention remain important issues because of population ageing, the increasing burden of chronic diseases and growing demand for primary and specialised nursing care. Identifying factors associated with professional identity among nursing interns is therefore important for optimising clinical education, supporting role transition and strengthening the future nursing workforce.

Several studies in China have examined factors associated with professional identity among nursing interns, but the findings remain scattered and have not been systematically synthesised. This study aimed to integrate the available evidence on factors associated with professional identity among nursing interns in China through a systematic review and meta-analysis. Factors reported in at least two studies were quantitatively pooled using Pearson correlation coefficients, whereas factors reported in only one study were synthesised descriptively. The findings may inform nursing education, clinical mentoring management and future research.

## Methods

This study was reported in accordance with the Preferred Reporting Items for Systematic Reviews and Meta-Analyses 2020 statement (PRISMA 2020) and the Meta-analysis of Observational Studies in Epidemiology (MOOSE) guidelines. Further details are provided in the supplementary materials. The protocol was registered in the International Prospective Register of Systematic Reviews (PROSPERO; registration number: CRD420261360963).

### Search strategy

A systematic search was conducted in the Cochrane Library, CINAHL, PubMed, Embase, Web of Science, SinoMed, China National Knowledge Infrastructure (CNKI), Wanfang Database and VIP Chinese Science and Technology Journal Database from database inception to 20 January 2026. Controlled vocabulary terms and free-text terms were combined, and the reference lists of the included studies were manually searched to identify additional eligible studies. The complete database-specific search strategies are provided in supplementary materials.

The Chinese search terms mainly included “nursing students”, “nursing interns”, “student nurses”, “professional identity”, “professional identification”, “influencing factors” and “associated factors”. The English search terms mainly included “nursing intern”, “nursing student”, “student nurse”, “professional identity”, “professional recognition”, “professional commitment”, “factors” and “correlates”. Boolean operators, including AND and OR, were used to combine search terms.

### Inclusion and exclusion criteria

#### Inclusion criteria

Studies were included if they met the following criteria:

1. The study design was cross-sectional.
2. The participants were nursing interns in China, defined as nursing students who had entered clinical internship during their nursing education but had not yet graduated or formally obtained registered nurse status.
3. The study examined professional identity among nursing interns and its associated factors.
4. Professional identity was measured using a professional identity scale or its derivative instruments, with acceptable reliability reported.

#### Exclusion criteria

Studies were excluded if they met any of the following criteria:

1. The publication was not written in Chinese or English.
2. The study was a duplicate publication.
3. The full text was unavailable or the data were incomplete.
4. The publication was a conference paper, abstract, review or other non-original study.
5. Pearson correlation analysis was not used in the statistical analysis.
6. The total Pearson correlation coefficient related to professional identity was not reported and could not be derived from the available data.
7. The participants did not meet the definition of nursing interns used in this study, or the study population was mixed and data for nursing interns could not be extracted separately.

### Study selection and data extraction

EndNote software was used to identify and remove duplicate records retrieved from the database searches, followed by manual verification of duplicate publications. Two reviewers independently screened the studies according to the predefined inclusion and exclusion criteria. Titles and abstracts were first screened to exclude clearly irrelevant records, and the full texts of potentially eligible studies were then assessed to determine final inclusion. Any disagreements between the two reviewers were resolved through discussion with a third reviewer. Data were independently extracted by two reviewers and recorded in Microsoft Excel. The extracted information included:

1. Basic information of the included studies, including first author, year of publication, study region, professional identity measurement instruments with acceptable reliability and validity, and associated factors;
2. Basic characteristics of the study population, including sample size and sampling method;
3. Overall Pearson correlation coefficients (r).

Variables with similar names but clear differences in conceptual definitions, measurement instruments or dimensional structures were not simply combined; instead, they were extracted and classified separately according to the definitions used in the original studies. Variables with consistent conceptual meanings and adequate comparability were combined for subsequent analysis.

### Quality assessment

The methodological quality of the included studies was assessed using the quality assessment criteria for cross-sectional studies recommended by the Agency for Healthcare Research and Quality (AHRQ) [9]. This tool consists of 11 items, each of which is rated as “yes”, “no” or “unclear”. A total score of 8–11 indicates high quality, 4–7 indicates moderate quality, and 0–3 indicates low quality. Quality assessment was performed independently by two reviewers. Any disagreements were resolved through discussion, and a third reviewer was consulted when necessary. The detailed quality assessment is presented in the supplementary materials.

### Statistical analysis

Meta-analysis was performed using RevMan 5.4. Pearson’s correlation coefficient (r) was used as the effect size. Because the sampling distribution of r is constrained by its value range and does not follow a normal distribution, the r values from individual studies were first transformed into Fisher’s z values before pooling to improve the stability of the estimates. The transformation formulas were as follows [10]:

$$z=\frac{1}{2}\mathrm{ln}\left(\frac{1+r}{1-r}\right); \:\mathrm{S}\mathrm{E}=\sqrt{\frac{1}{\mathrm{n}-3}}; \:{\mathrm{V}}_{\mathrm{z}}=\frac{1}{\mathrm{n}-3}$$

where n represents the sample size.The inverse variance method was used to pool Fisher’s z values and their 95% confidence intervals (95% CIs). The pooled Fisher’s z values and corresponding 95% CIs were then back-transformed into Pearson’s correlation coefficients (r) to facilitate interpretation. The back-transformation formula was:$\mathrm{r}=\frac{{\mathrm{e}}^{2\mathrm{z}}-1}{{\mathrm{e}}^{2\mathrm{z}}+1}$。Heterogeneity was assessed using the I² statistic. A random-effects model was applied when I² ≥ 50%; otherwise, a fixed-effect model was used. For associated factors that could not be quantitatively pooled because of the limited number of studies or insufficient comparable data, a descriptive synthesis was performed.

## Results

### Study selection

**Fig. 1:**
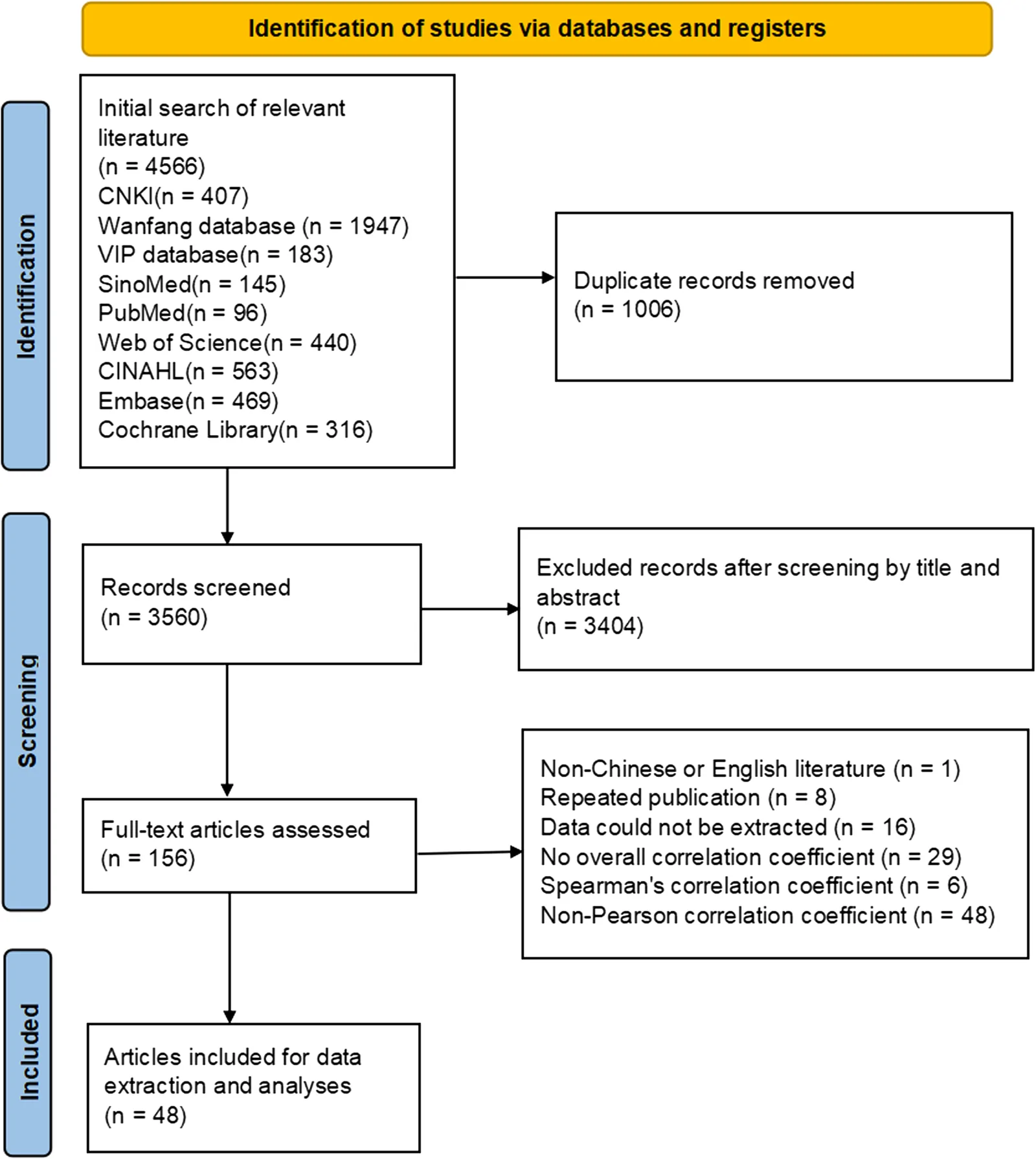
PRISMA flow diagram of the selection process of studies

A total of 4,566 records were identified through searches of CNKI, Wanfang Database, VIP Database, SinoMed, PubMed, CINAHL, Web of Science, Embase and the Cochrane Library. After removing 1,006 duplicate records, 3,560 records remained. Following screening of titles and abstracts, 3,404 irrelevant records were excluded. The full texts of the remaining 156 articles were assessed for eligibility, of which 108 were excluded according to the inclusion and exclusion criteria. Finally, 48 studies were included in this review. The study selection process is shown in Fig. 1.

### Basic characteristics and quality assessment of the included studies

All 48 included studies were cross-sectional studies, comprising 41 Chinese-language publications and seven English-language publications. A total of 42 associated factors were identified; 12 factors reported in at least two studies were included in the quantitative meta-analysis, and the remaining 30 were synthesised descriptively. The total sample size was 16,459 participants. The quality assessment scores of the included studies ranged from 3 to 9, indicating that most studies were of moderate methodological quality. The basic characteristics and quality assessment results of the included studies are presented in Table 1.

**Table 1:** Basic characteristics and quality assessment of included studies

No.	First author / Year	Region	Sample size	Evaluation tools	Associated factors	Total score	Literature level
S1	Liu 2023 [11]	Hainan, etc.	185	PIQNS	①	7	medium
S2	Lei 2024 [12]	Hengyang	469	PIQNS	①②	6	medium
S3	Li 2022 [13]	Shanghai, etc.	293	NIS	①	5	medium
S4	Wang 2018 [14]	Changsha	367	PIQNS	③	5	medium
S5	Su 2024 [15]	Inner Mongolia, etc.	301	PIQNS	③	7	medium
S6	Yu 2017 [16]	Xiaogan	278	NIS-R	④	4	medium
S7	Zhang 2016 [17]	Tianjin	288	NIS	④	7	medium
S8	Hu 2025 [18]	Zhengzhou	300	NIS	⑤⑥	5	medium
S9	Sun 2016 [19]	Xiaogan	200	NIS-R	⑦	4	medium
S10	He 2021 [20]	Jinan	252	NIS	⑧⑨	6	medium
S11	Wang 2023 [21]	Shanghai, etc.	232	PIQNS	⑧⑩	8	high
S12	Xu 2019 [22]	Zhengzhou	133	PIQNS	⑧⑯	6	medium
S13	Zhang 2022 [23]	Zhengzhou	649	PIQNS	⑪	7	medium
S14	Luo 2025 [24]	Kunming	98	NIS	⑫	6	medium
S15	Guo 2017 [25]	Tai’an	258	NIS	①	4	medium
S16	Sun 2023 [26]	Zhengzhou	847	NIS-B	⑬⑭⑮	9	high
S17	Wang 2021 [27]	Changzhi	116	PIQNS	⑯	3	low
S18	Xiao 2016 [28]	Changsha	651	PIQNS	⑯	7	medium
S19	Sun 2020 [29]	Beijing	537	PIQNS	⑯	7	medium
S20	Shi 2021 [30]	Dalian	144	PIQNS	⑯⑰	7	medium
S21	Li 2019 [31]	Luoyang	145	PIQNS	⑱	6	medium
S22	Peng 2022 [32]	Henan	175	PIQNS	⑲	7	medium
S23	Liu 2020 [33]	Tai’an	414	PIQNS	⑳	4	medium
S24	Li 2024 [34]	Shanghai	207	PIQNS	㉑	7	medium
S25	Liu 2015 [35]	Shanghai	200	PIQNS	⑭	5	medium
S26	Dou 2018 [36]	Zhengzhou	595	NIS-B	⑭	6	medium
S27	Wang 2024 [37]	Guiyang	220	PIQNS	②	6	medium
S28	Ou 2020 [38]	Hengyang	140	NIS	㉒	6	medium
S29	Wang 2022 [39]	Zhengzhou	277	PIQNS	㉓	7	medium
S30	Yuan 2021 [40]	Chongqing	295	PIQNS	㉔㉕	6	medium
S31	Zhang 2024 [41]	Changsha, Xiangtan	331	PIQNS	㉔	5	medium
S32	Zhao 2022 [42]	Zibo	273	PIQNS	㉖	8	high
S33	Xu 2023 [43]	Hangzhou	204	PIQNS	㉗	7	medium
S34	Nong 2022 [44]	Nanning	84	PIQNS	㉘	6	medium
S35	Wang 2025 [45]	Beijing	322	PIQNS	㉙㉚	7	medium
S36	He 2022 [46]	Sichuan	425	PIQNS	⑯	8	high
S37	Yu 2024 [47]	Hangzhou	248	PIQNS	㉛	6	medium
S38	Xu 2023 [48]	Suzhou	127	PIQNS	㉜	6	medium
S39	Fu 2019 [49]	Jining, Jinan	282	NIS	㉝	7	medium
S40	Meng 2024 [50]	Luzhou	370	NIS	㉞	7	medium
S41	Jiang 2025 [51]	Bengbu	252	PIQNS	⑧㉔	7	medium
S42	Lin 2025 [52]	Hangzhou	377	PIQNS	④⑤㉟	7	medium
S43	Sun 2016 [53]	China	474	PIQNS	㉟	6	medium
S44	Lin 2023 [54]	Fuzhou	222	PIQNS	⑧㊱	7	medium
S45	Zhang 2025 [55]	Sichuan	1050	PIQNS	⑬㊲	9	high
S46	Yao 2021 [56]	China	887	PIQNS	⑯㊳	8	high
S47	Yang 2025 [57]	Changsha	791	PIQNS	⑱㊴㊵	5	medium
S48	Zhu 2024 [58]	Southwest and Central China	474	PIQNS	㊶㊷	6	medium

Note: ①humanistic caring ability; ②medical narrative competence;③perceived hospital care climate; ④clinical learning environment; ⑤perceived professional benefit; ⑥self-directed learning ability; ⑦learning burnout; ⑧psychological resilience; ⑨psychological stress; ⑩mental health risk; ⑪psychological status; ⑫psychological violence; ⑬anxiety; ⑭perceived teacher support; ⑮benefit finding; ⑯: professional self-efficacy; ⑰professional role model; ⑱clinical practice behaviour; ⑲self-transcendence ability; ⑳: learning motivation; ㉑work engagement; ㉒internship satisfaction; ㉓alienation; ㉔social support; ㉕professional quality of life; ㉖transition shock; ㉗work readiness; ㉘post competency of clinical preceptors; ㉙moral sensitivity; ㉚professional dedication; ㉛professional ethical attitude; ㉜workplace bullying; ㉝adversity quotient; ㉞professional interest; ㉟role stress; ㊱learning engagement; ㊲clinical sense of belonging; ㊳self-efficacy; ㊴clinical communication ability; ㊵ professional values; ㊶ motivation; ㊷ innovation ability

### Meta-analysis results of factors associated with professional identity among nursing interns

The meta-analysis results are shown in Table 2. Factors positively associated with professional identity among nursing interns included humanistic caring ability, clinical learning environment, psychological resilience, professional self-efficacy, perceived professional benefit, perceived teacher caring, social support, medical narrative competence, and perceived hospital caring climate. Role stress and anxiety were negatively associated with professional identity. The association between clinical practice behaviour and professional identity was not statistically significant. Thirty factors were reported in only one study each and therefore were not statistically pooled; these factors were synthesised descriptively.

**Table 2:** Results of meta‑analysis on factors associated with professional identity among nursing interns

Associated factors	No. of studies	Heterogeneity		Model	Meta‑analysis results		
		I^2^(%)	*P*		Fisher’s z and 95%CI	*P*	Summary *r* and 95%CI
A	4	99	< 0.00001	R	1.02(0.52, 1.53)	< 0.0001	0.77 (0.48, 0.91)
B	3	98	< 0.00001	R	0.59(0.18, 1.01)	0.005	0.53 (0.18, 0.77)
C	5	85	< 0.0001	R	0.57(0.41, 0.72)	< 0.00001	0.52 (0.39, "0.62")
D	7	96	< 0.00001	R	0.73(0.54, 0.91)	< 0.00001	0.62 ( 0.49, 0.72)
E	3	90	< 0.0001	R	0.38(0.22, 0.55)	< 0.00001	0.36 (0.22, 0.50)
F	2	78	0.03	R	0.48(0.32, 0.65)	< 0.00001	0.45 (0.31, 0.57)
G	2	0	0.53	F	0.76(0.69, 0.84)	< 0.00001	0.64 (0.60, 0.69)
H	2	96	< 0.00001	R	0.44(-0.03, 0.92)	0.07	0.41 (-0.03, 0.73)
I	2	8	0.30	F	0.73(0.65, 0.80)	< 0.00001	0.62 (0.57, 0.66)
J	3	91	< 0.0001	R	0.58(0.35,0.80)	< 0.00001	0.52 (0.34, 0.66)
K	2	0	0.39	F	-0.33(-0.40, -0.26)	< 0.00001	-0.32 (-0.38, -0.25)
L	2	0	0.61	F	-0.14(-0.19, -0.10)	< 0.00001	-0.14 (-0.19, -0.10)

Note: A: humanistic caring ability; B: clinical learning environment; C: psychological resilience; D: professional self-efficacy; E: perceived teacher caring; F: perceived hospital caring climate; G: medical narrative competence; H: clinical practice behaviour; I: perceived professional benefit; J: social support; K: role stress; L: anxiety; R: random-effects model; F: fixed-effect model

### Association between humanistic caring ability and professional identity

**Fig. 2:**
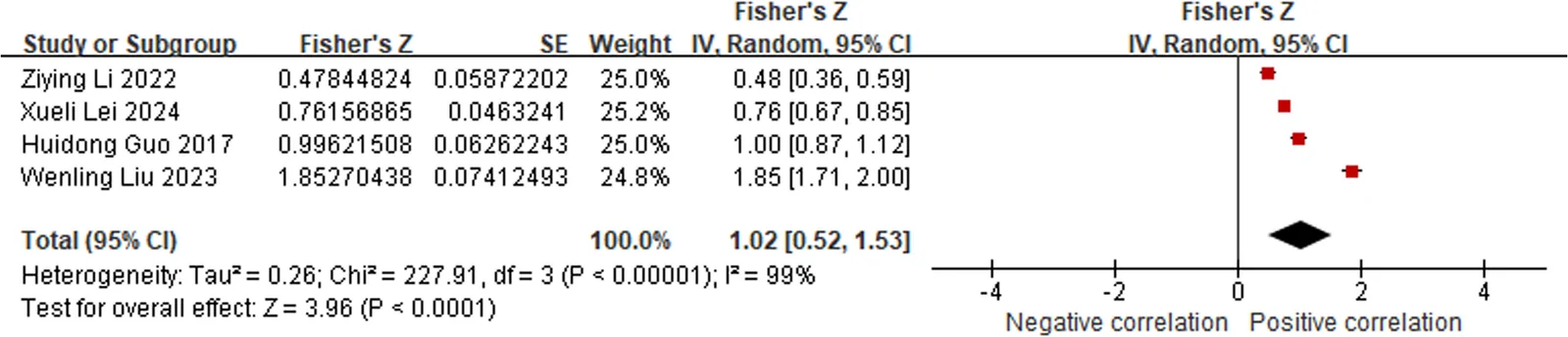
Fisher’s Z forest plot for humanistic caring ability and professional identity

As shown in Fig. 2, four studies were included in the meta-analysis. The pooled Fisher’s z value was 1.02 (95% CI: 0.52–1.53, *P* < 0.0001), indicating a statistically significant association. After back-transformation, the pooled correlation coefficient was *r* = 0.77 (95% CI: 0.48–0.91, *P* < 0.0001), suggesting that humanistic caring ability was positively associated with professional identity among nursing interns. Substantial heterogeneity was observed across studies; therefore, a random-effects model was used for the meta-analysis.

### Association between clinical learning environment and professional identity

**Fig. 3:**
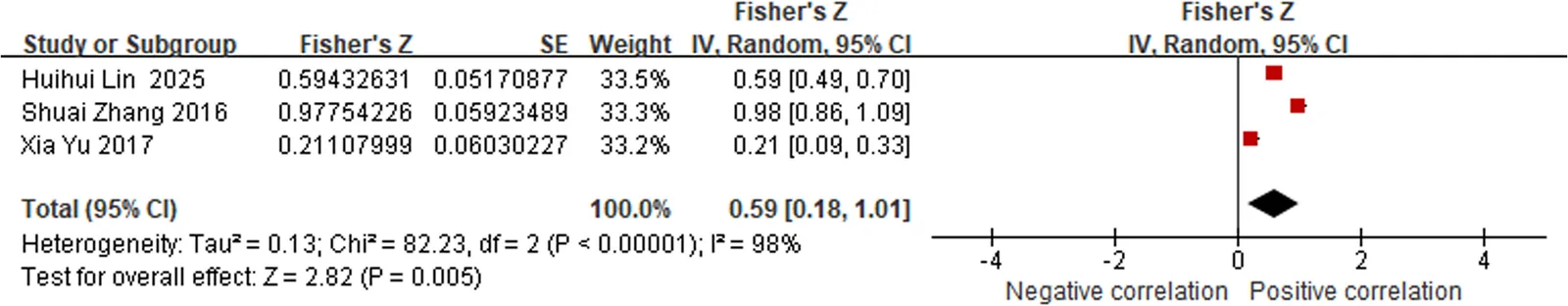
Fisher’s Z forest plot for clinical learning environment and professional identity

As shown in Fig. 3, the pooled Fisher’s z value was 0.59 (95% CI: 0.18–1.01, *P* = 0.005), indicating a statistically significant association. After back-transformation, the pooled correlation coefficient was *r* = 0.53 (95% CI: 0.18–0.77, *P* = 0.005), suggesting that the clinical learning environment was positively associated with professional identity among nursing interns. Substantial heterogeneity was observed across studies; therefore, a random-effects model was used for the meta-analysis.

### Association between psychological resilience and professional identity

**Fig. 4:**
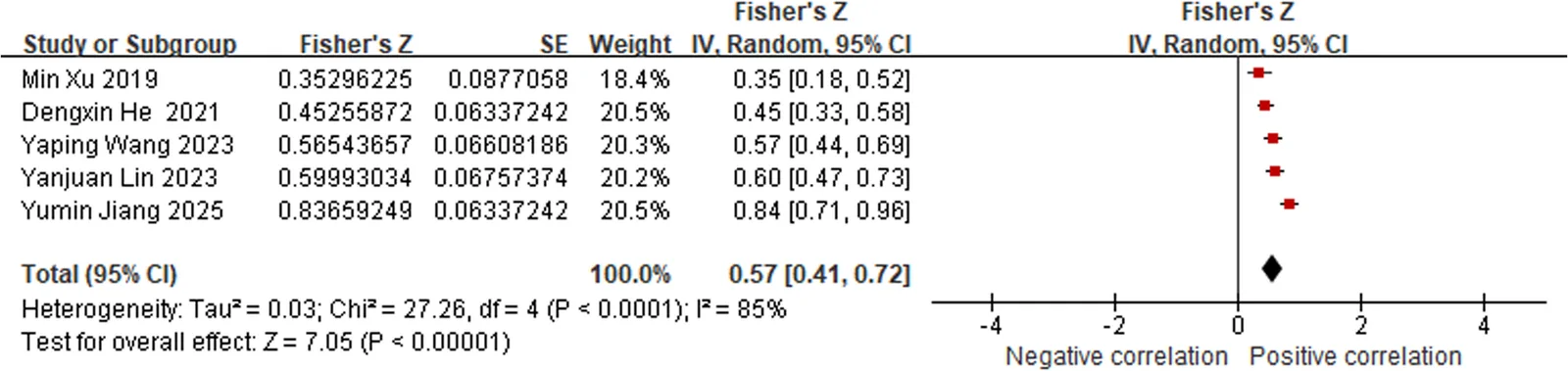
Fisher’s Z forest plot for psychological resilience and professional identity

As shown in Fig. 4, the pooled Fisher’s z value was 0.57 (95% CI: 0.41–0.72, *P* < 0.00001), indicating a statistically significant association. After back-transformation, the pooled correlation coefficient was *r* = 0.52 (95% CI: 0.39–0.62, *P* < 0.00001), suggesting that psychological resilience was positively associated with professional identity among nursing interns. Substantial heterogeneity was observed across studies; therefore, a random-effects model was used for the meta-analysis.

### Association between professional self-efficacy and professional identity

**Fig. 5:**
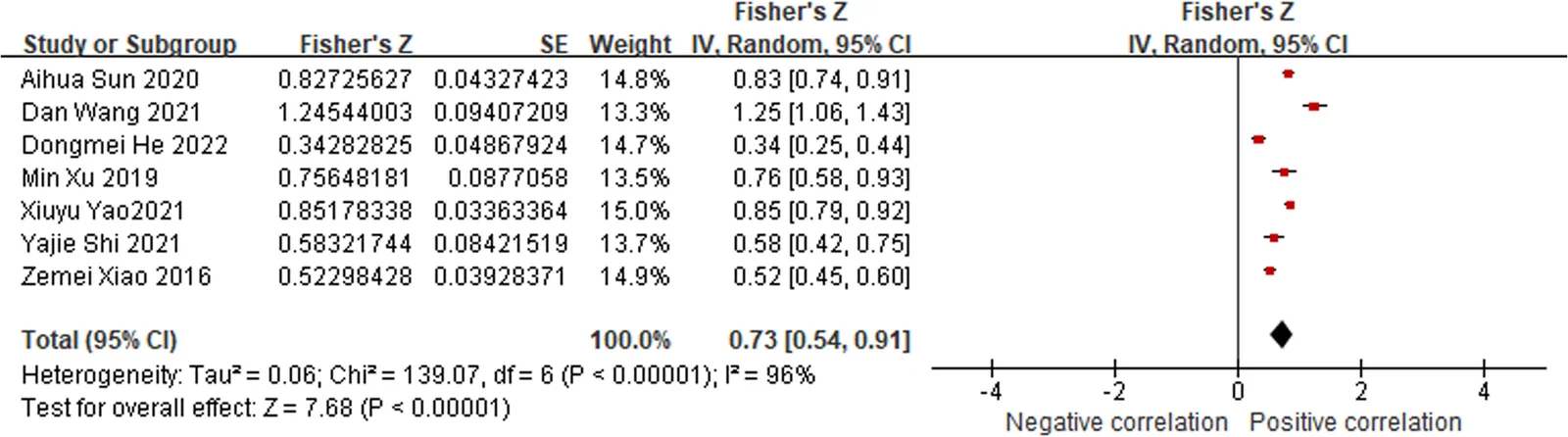
Fisher’s Z forest plot for professional self-efficacy and professional identity

As shown in Fig. 5, the pooled Fisher’s z value was 0.73 (95% CI: 0.54–0.91, *P* < 0.00001), indicating a statistically significant association. After back-transformation, the pooled correlation coefficient was *r* = 0.62 (95% CI: 0.49–0.72, *P* < 0.00001), suggesting that professional self-efficacy was positively associated with professional identity among nursing interns. Substantial heterogeneity was observed across studies; therefore, a random-effects model was used for the meta-analysis.

### Association between perceived teacher caring and professional identity

**Fig. 6:**
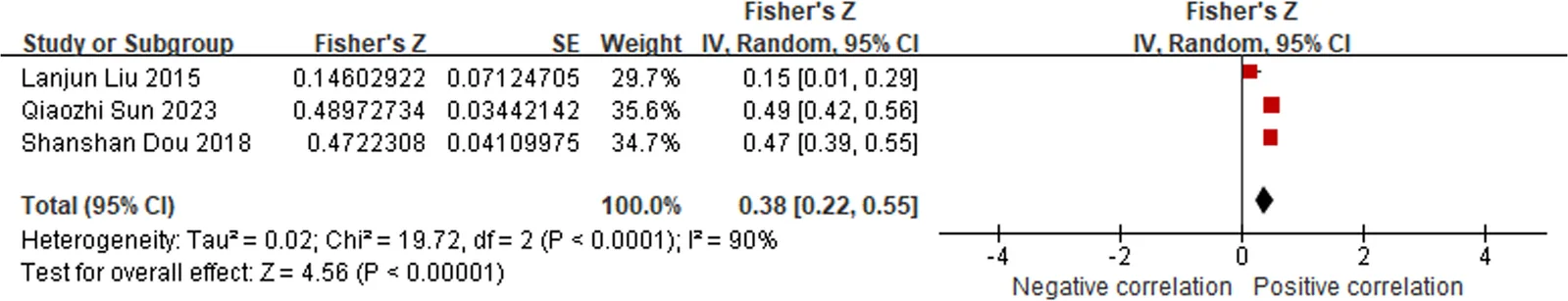
Fisher’s Z forest plot for perceived teacher caring and professional identity

As shown in Fig. 6, the pooled Fisher’s z value was 0.38 (95% CI: 0.22–0.55, *P* < 0.00001), indicating a statistically significant association. After back-transformation, the pooled correlation coefficient was *r* = 0.36 (95% CI: 0.22–0.50, *P* < 0.00001), suggesting that perceived teacher caring was positively associated with professional identity among nursing interns. Substantial heterogeneity was observed across studies; therefore, a random-effects model was used for the meta-analysis.

### Association between perceived hospital caring climate and professional identity

**Fig. 7:**
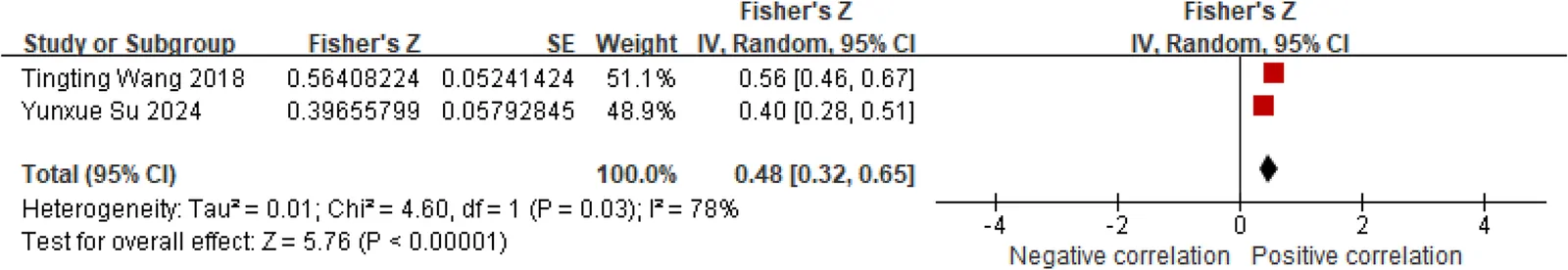
Fisher’s Z forest plot for perceived hospital caring climate and professional identity

As shown in Fig. 7, the pooled Fisher’s z value was 0.48 (95% CI: 0.32–0.65, *P* < 0.00001), indicating a statistically significant association. After back-transformation, the pooled correlation coefficient was *r* = 0.45 (95% CI: 0.31–0.57, *P* < 0.00001), suggesting that perceived hospital caring climate was positively associated with professional identity among nursing interns. Substantial heterogeneity was observed across studies; therefore, a random-effects model was used for the meta-analysis.

### Association between medical narrative competence and professional identity

**Fig. 8:**
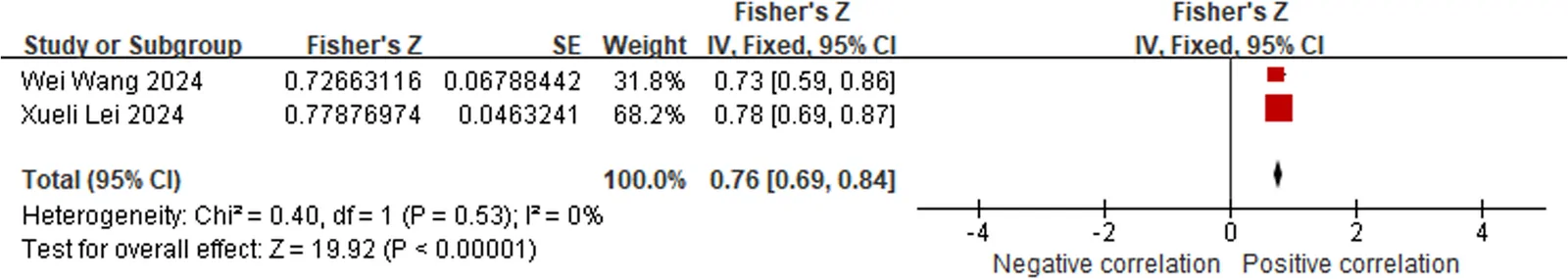
Fisher’s Z forest plot for medical narrative competence and professional identity

As shown in Fig. 8, the pooled Fisher’s z value was 0.76 (95% CI: 0.69–0.84, *P* < 0.00001), indicating a statistically significant association. After back-transformation, the pooled correlation coefficient was *r* = 0.64 (95% CI: 0.60–0.69, *P* < 0.00001), suggesting that medical narrative competence was positively associated with professional identity among nursing interns. No substantial heterogeneity was observed across studies; therefore, a fixed-effect model was used for the meta-analysis.

### Association between perceived professional benefit and professional identity

**Fig. 9:**
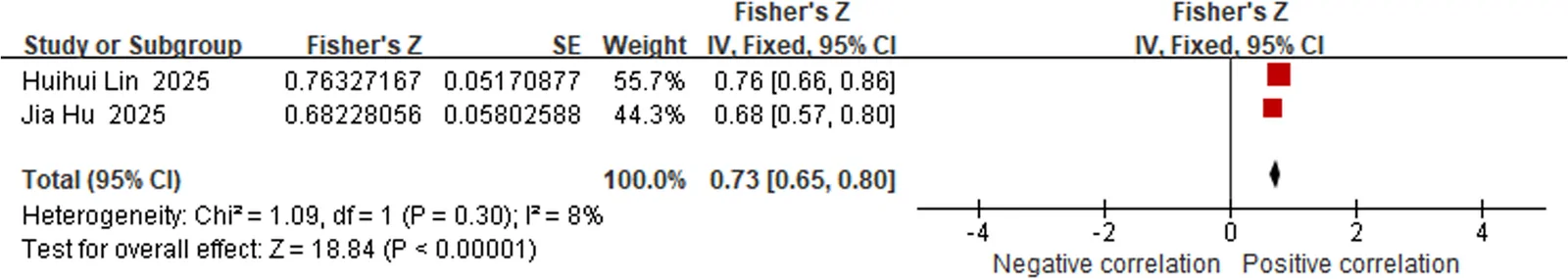
Fisher’s Z forest plot for perceived professional benefit and professional identity

As shown in Fig. 9, the pooled Fisher’s z value was 0.73 (95% CI: 0.65–0.80, *P* < 0.00001), indicating a statistically significant association. After back-transformation, the pooled correlation coefficient was *r* = 0.62 (95% CI: 0.57–0.66, *P* < 0.00001), suggesting that perceived professional benefit was positively associated with professional identity among nursing interns. Low heterogeneity was observed across studies; therefore, a fixed-effect model was used for the meta-analysis.

### Association between clinical practice behaviour and professional identity

**Fig. 10:**
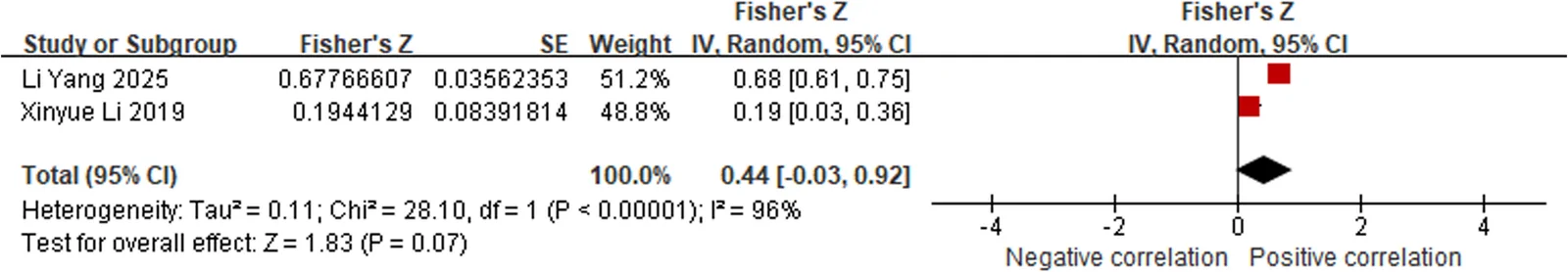
Fisher’s Z forest plot for clinical practice behaviour and professional identity

As shown in Fig. 10, the pooled Fisher’s z value was 0.44 (95% CI: -0.03–0.92, *P* = 0.07), indicating that the association was not statistically significant. After back-transformation, the pooled correlation coefficient was *r* = 0.41 (95% CI: -0.03–0.73, *P* = 0.07), suggesting that clinical practice behaviour was not significantly associated with professional identity among nursing interns. Substantial heterogeneity was observed across studies; therefore, a random-effects model was used for the meta-analysis.

### Association between social support and professional identity

**Fig. 11:**
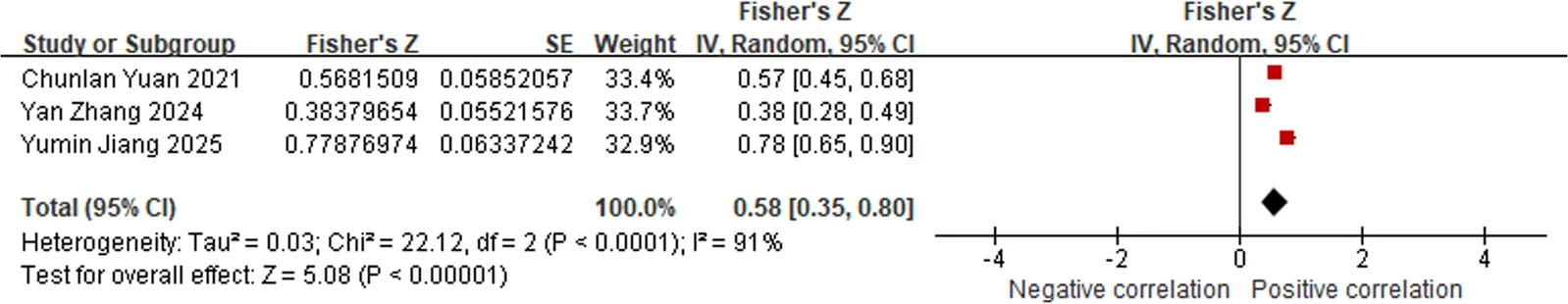
Fisher’s Z forest plot for social support and professional identity

As shown in Fig. 11, the pooled Fisher’s z value was 0.58 (95% CI: 0.35–0.80, *P* < 0.00001), indicating a statistically significant association. After back-transformation, the pooled correlation coefficient was *r* = 0.52 (95% CI: 0.34–0.66, *P* < 0.00001), suggesting that social support was positively associated with professional identity among nursing interns. Substantial heterogeneity was observed across studies; therefore, a random-effects model was used for the meta-analysis.

### Association between role stress and professional identity

**Fig. 12:**
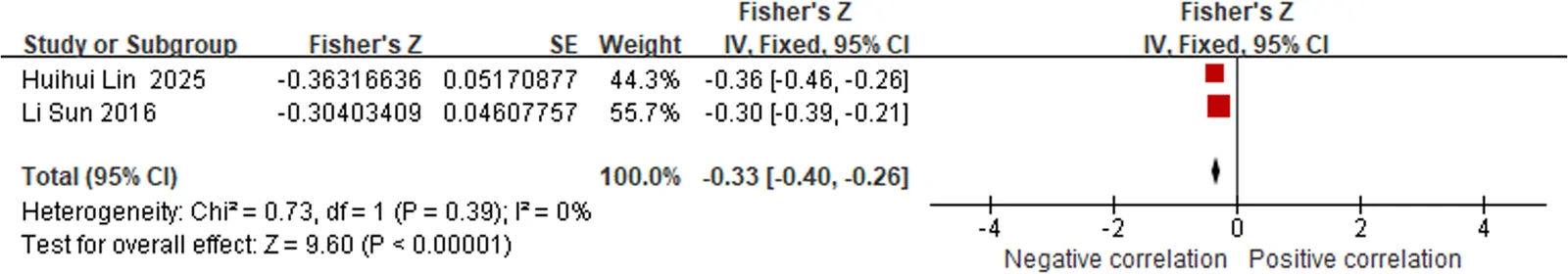
Fisher’s Z forest plot for role stress and professional identity

As shown in Fig. 12, the pooled Fisher’s z value was − 0.33 (95% CI: -0.40 to -0.26, *P* < 0.00001), indicating a statistically significant association. After back-transformation, the pooled correlation coefficient was *r* = -0.32 (95% CI: -0.38 to -0.25, *P* < 0.00001), suggesting that role stress was negatively associated with professional identity among nursing interns. No substantial heterogeneity was observed across studies; therefore, a fixed-effect model was used for the meta-analysis.

### Association between anxiety and professional identity

**Fig. 13:**
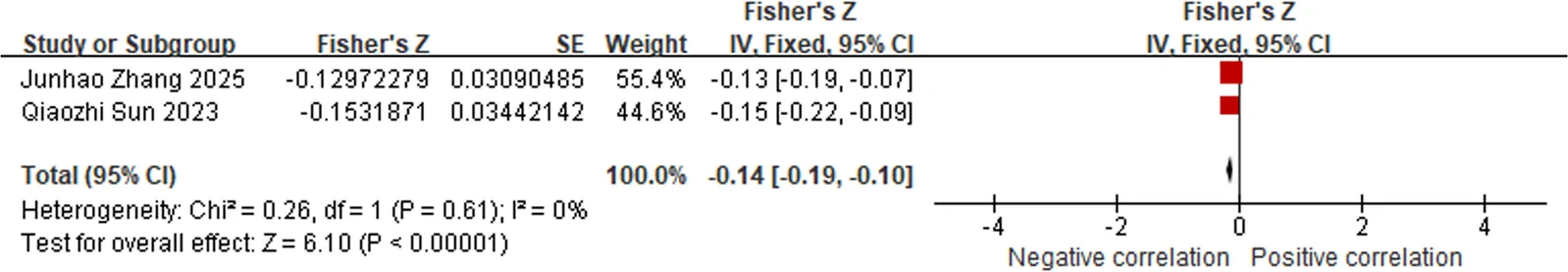
Fisher’s Z forest plot for anxiety and professional identity

As shown in Fig. 13, the pooled Fisher’s z value was − 0.14 (95% CI: -0.19 to -0.10, *P* < 0.00001), indicating a statistically significant association. After back-transformation, the pooled correlation coefficient was *r* = -0.14 (95% CI: -0.19 to -0.10, *P* < 0.00001), suggesting that anxiety was negatively associated with professional identity among nursing interns. No substantial heterogeneity was observed across studies; therefore, a fixed-effect model was used for the meta-analysis.

### Publication bias and sensitivity analyses

#### Assessment of publication bias

**Fig. 14:**
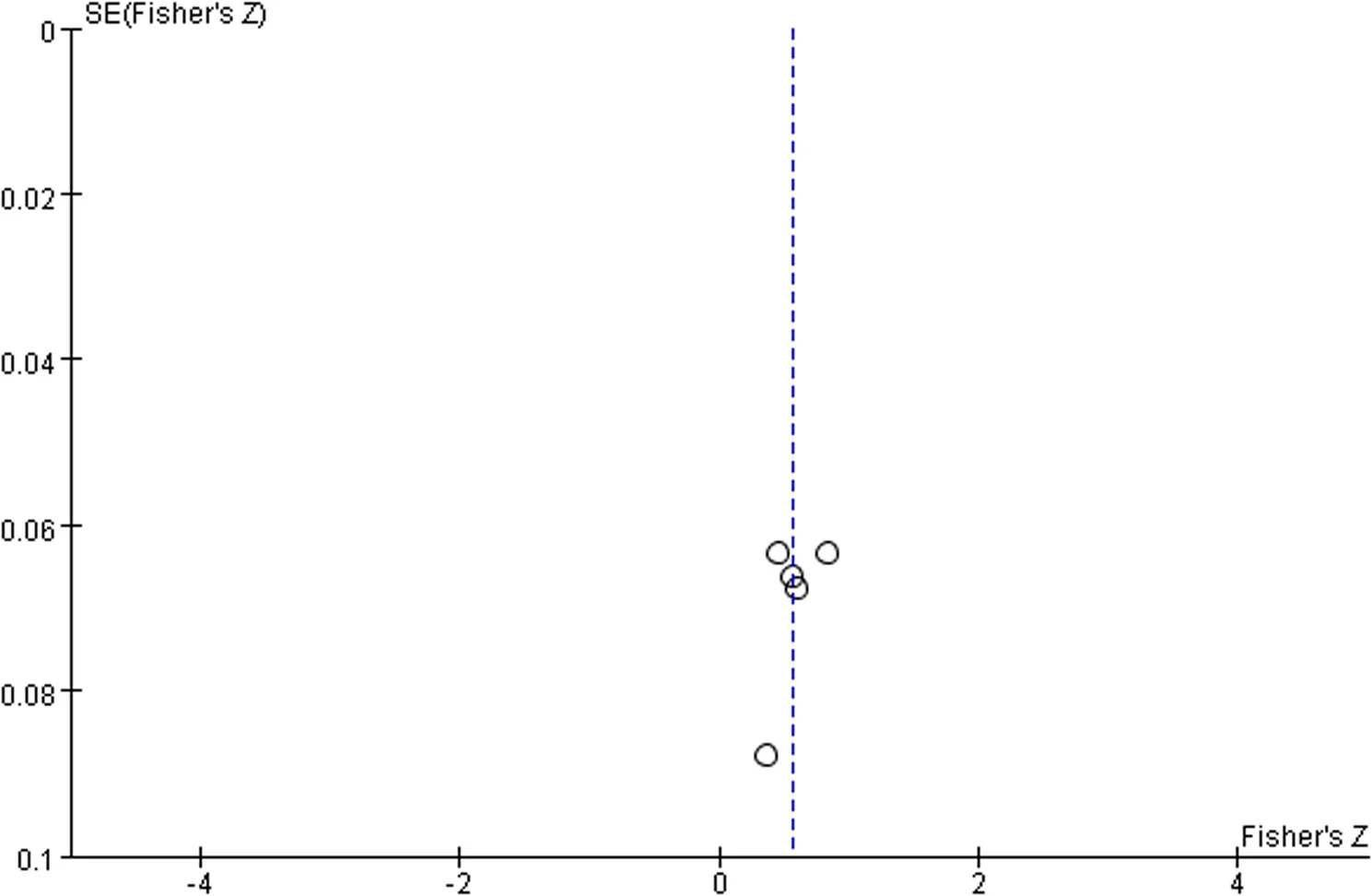
Funnel plot for professional self-efficacy and professional identity correlation analysis

**Fig. 15:**
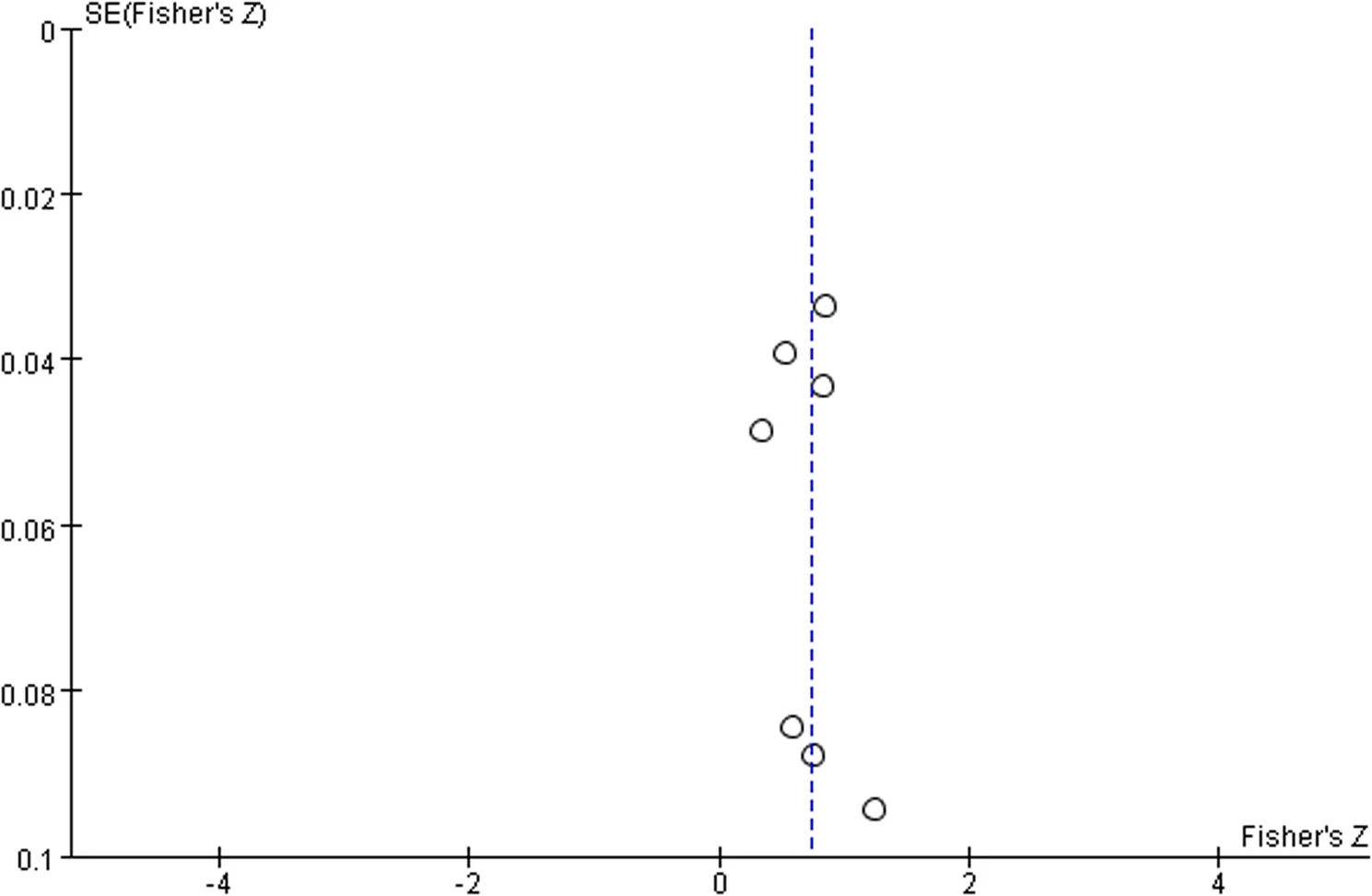
Funnel plot for psychological resilience and professional identity correlation analysis

Exploratory assessment of publication bias was performed only for factors with a relatively larger number of included studies, including professional self-efficacy and professional identity, with seven studies, and psychological resilience and professional identity, with five studies. The funnel plot for professional self-efficacy and professional identity is shown in Fig. 14, and the funnel plot for psychological resilience and professional identity is shown in Fig. 15. Visual inspection of the funnel plots showed no obvious asymmetry in either analysis. However, because fewer than 10 studies were included in each analysis, the ability of funnel plots to detect publication bias was limited. Therefore, the results of the publication bias assessment should be interpreted with caution.

### Sensitivity analysis

Leave-one-out sensitivity analyses were conducted for the associations between professional self-efficacy and professional identity, and between psychological resilience and professional identity. The results showed that the direction of the pooled effect did not change after sequentially excluding each individual study, and the findings remained statistically significant, suggesting that the direction of the pooled associations was relatively stable. Notably, in the analysis of psychological resilience, heterogeneity decreased after excluding Jiang 2025, indicating that this individual study may have contributed to heterogeneity. Therefore, these results should still be interpreted cautiously. Detailed results are presented in Tables 3 and 4 .

**Table 3:** Sensitivity analysis of the correlation between professional self-efficacy and professional identity

Program	Z-value	95%CI	I² (%)
Yao 2021	0.71	[0.49, 0.92]	95
He 2022	0.79	[0.62, 0.96]	94
Sun 2020	0.71	[0.49, 0.93]	96
Shi 2021	0.75	[0.54, 0.96]	96
Xu 2019	0.72	[0.52, 0.93]	96
Wang 2021	0.65	[0.47, 0.83]	95
Xiao 2016	0.75	[0.55, 0.98]	96

**Table 4:** Sensitivity analysis of the correlation between psychological resilience and professional identity

Program	Z-value	95%CI	I² (%)
Lin 2023	0.56	[0.35, 0.76]	89
Wang 2023	0.56	[0.36, 0.77]	89
He 2021	0.59	[0.41, 0.78]	86
Xu 2019	0.61	[0.45, 0.78]	85
Jiang 2025	0.50	[0.40, 0.60]	54

### Subgroup analysis

Because the number of studies included for each associated factor was limited, exploratory subgroup analyses were conducted only for the associations between professional self-efficacy and professional identity, and between psychological resilience and professional identity.

For the association between professional self-efficacy and professional identity, subgroup analyses were performed according to publication year, measurement instrument and sample size (Table 5). When grouped by publication year, the pooled Fisher’s z values for the 2016–2020, 2021 and 2022 subgroups were 0.70 (95% CI: 0.48–0.92), 0.89 (95% CI: 0.60–1.18) and 0.34 (95% CI: 0.25–0.44), respectively, indicating numerical differences in pooled effect sizes across publication-year subgroups. When grouped by measurement instrument, the subgroup using the Hao Yufang scale had a higher pooled Fisher’s z value of 0.94 (95% CI: 0.68–1.19), while the subgroups using the German General Self-Efficacy Scale and the Peng Yongxin scale had pooled Fisher’s z values of 0.60 (95% CI: 0.10–1.10) and 0.53 (95% CI: 0.46–0.60), respectively. When grouped by sample size, all subgroup pooled effect sizes were positive, but no consistent trend was observed. For the association between psychological resilience and professional identity, subgroup analyses were performed according to publication year, measurement instrument and region (Table 6). When grouped by publication year, the pooled Fisher’s z values for the 2019–2021 and 2022–2025 subgroups were 0.42 (95% CI: 0.32–0.52) and 0.67 (95% CI: 0.50–0.84), respectively, indicating numerical differences in pooled effect sizes across publication-year subgroups. When grouped by measurement instrument, the pooled Fisher’s z values for the Brief Resilience Scale, CD-RISC and revised CD-RISC subgroups were 0.45 (95% CI: 0.33–0.58), 0.57 (95% CI: 0.44–0.69) and 0.60 (95% CI: 0.34–0.86), respectively, with positive pooled effect sizes in all subgroups. When grouped by region, the pooled Fisher’s z value was 0.58 in both the southern and northern China subgroups, suggesting no clear regional difference between these subgroups.

**Table 5:** Subgroup analysis of the correlation between professional self-efficacy and professional identity

Variable	Literature volume	Merged Z-value	95% CI	Heterogeneity (I²)%
Year				
2016–2020	3	0.70	[0.48,0.92]	93
2021	3	0.89	[0.60,1.18]	93
2022	1	0.34	[0.25,0.44]	Not applicable
Measurement Tool				
Haoyufang Scale	3	0.94	[0.68,1.19]	89
German GSES Scale	2	0.60	[0.10,1.10]	89
Peng-Yongxin Scale	2	0.53	[0.46,0.60]	0
Sample Size				
100-200	3	0.86	[0.48, .24]	93
200-600	2	0.59	[0.11,1.06]	98
＞600	2	0.69	[0.37,1.01]	98
Total	7	0.73	[0.54,0.91]	96

**Table 6:** Subgroup analysis of the correlation between psychological resilience and professional identity

Variable	Literature volume	Merged Z-value	95% CI	Heterogeneity (I²)%
Year				
2019–2021	2	0.42	[0.32, 0.52]	0
2022–2025	3	0.67	[0.50, 0.84]	81
Measurement Tool				
Simple Psychological Resilience Scale	1	0.45	[0.33, 0.58]	Not applicable
CD-RISC	1	0.57	[0.44, 0.69]	Not applicable
CD-RISC-R	3	0.60	[0.34, 0.86]	90
Region				
South	2	0.58	[0.49, 0.67]	0
North	3	0.58	[0.51, 0.66]	93
Total	5	0.57	[0.41, 0.72]	85

### Descriptive synthesis of factors reported in only one study

In addition to the 12 associated factors included in the meta-analysis, the remaining 30 factors were each reported in only one study. Owing to the insufficient number of relevant studies and differences in the conceptual definitions, measurement instruments and dimensional structures of some variables, quantitative pooling was not performed. These factors were therefore synthesised descriptively.

### Individual psychological and competence-related factors

This category included mental health risk [21], psychological status [23], psychological violence [24], psychological stress [20], adversity quotient [49], clinical communication competence [57], self-transcendence ability [32], self-efficacy [56], innovation ability [58], self-directed learning ability [18] and work readiness [43]. These factors mainly reflected nursing interns’ psychological status, individual adaptability and professional competence. Findings from individual studies suggested that these factors may be associated with professional identity.

### Professional cognition, attitudes and internship experience-related factors

This category included learning motivation [33], motivation [58], professional values [57], professional ethics [45], professional dedication [45], professional ethical attitude [47], professional interest [50], benefit finding [26], work engagement [34], learning engagement [54], internship satisfaction [38], alienation [39], professional quality of life [40], transition shock [42], clinical sense of belonging [55] and learning burnout [19]. These factors mainly reflected nursing interns’ cognitive evaluation of the nursing profession, emotional attitudes and subjective experiences during clinical internship. Findings from individual studies suggested that these factors may be associated with professional identity.

### Educational support and workplace environment-related factors

This category included professional role model [30], workplace bullying [48] and clinical preceptors’ post competence [44]. These factors mainly reflected the support that nursing interns received in clinical teaching and workplace settings, or their exposure to negative workplace environments. Findings from individual studies suggested that these factors may be associated with professional identity.

As all 30 factors were reported in only one study, they lacked repeated validation. Therefore, the direction and strength of their associations with professional identity only reflected the findings of the original studies and could not be considered stable evidence. Further high-quality studies are needed to verify these associations.

### Demographic characteristics and internship-related background factors not included in the quantitative synthesis

The included studies suggested that professional identity among nursing interns may be associated with background factors such as professional choice, future career intention, family occupational environment, learning experience and perceptions of career prospects. In contrast, the roles of general demographic characteristics, such as sex, age, only-child status and place of origin, appeared to be relatively inconsistent. Because these findings were mainly derived from group comparisons, analysis of variance or regression analyses in individual studies, and because variable classification and reporting methods varied substantially across studies, these conclusions require further validation.

## Discussion

### Main findings

This meta-analysis systematically synthesised the available evidence on factors associated with professional identity among nursing interns in China. The findings showed that professional identity among nursing interns was associated with multiple domains, including individual psychological resources, professional competence, supportive clinical and educational environments, caring support, professional meaning-related experiences and negative psychological states.

Among the factors included in the meta-analysis, humanistic caring ability showed the strongest pooled correlation with professional identity (*r* = 0.77, 95% CI: 0.48–0.91), followed by medical narrative competence (*r* = 0.64, 95% CI: 0.60–0.69), professional self-efficacy (*r* = 0.62, 95% CI: 0.49–0.72) and perceived professional benefit (*r* = 0.62, 95% CI: 0.55–0.66). Psychological resilience (*r* = 0.52, 95% CI: 0.39–0.62), clinical learning environment (*r* = 0.53, 95% CI: 0.18–0.77) and social support (*r* = 0.52, 95% CI: 0.34–0.66) also showed moderate positive associations with professional identity. In contrast, role stress (*r* = -0.32, 95% CI: -0.38 to -0.25) and anxiety (*r* = -0.14, 95% CI: -0.19 to -0.10) were negatively associated with professional identity. The association between clinical practice behaviour and professional identity was not statistically significant (*r* = 0.41, 95% CI: -0.03–0.73, *P* = 0.07).

Overall, these findings suggest that professional identity among nursing interns is related to multiple domains rather than a single factor, but is related to multiple domains, including individual psychological resources, clinical educational environments and the construction of professional meaning. These findings may provide evidence for nursing educators and clinical managers to understand the development of professional identity among nursing interns from a multidimensional perspective.

### Individual psychological resources and professional identity

Professional self-efficacy (*r* = 0.62, 95% CI: 0.49–0.72) and psychological resilience (*r* = 0.52, 95% CI: 0.39–0.62) were moderately and positively associated with professional identity among nursing interns, suggesting that individual psychological resources may be important for understanding professional identity development.

Previous studies have indicated that nursing interns with higher professional self-efficacy are more likely to believe that they can complete nursing tasks, cope with clinical challenges and adapt to role transition, which may be related to more positive professional evaluation and stronger professional belonging [27]. Nursing interns with higher psychological resilience may be better able to regulate negative emotions, maintain professional beliefs and reduce withdrawal tendencies in high-pressure and complex clinical environments [59]. Thus, professional identity may reflect not only recognition of nursing professional values, but also interns’ perceptions of their competence and clinical adaptability.

These findings suggest that professional identity development should not rely solely on professional ideal education. Structured feedback, progressive clinical tasks, positive mentoring experiences and coping skills training may help support professional self-efficacy and psychological resilience. However, substantial heterogeneity was observed in both analyses, which may be related to differences in sample sources, internship stages, measurement instruments and hospital contexts. Therefore, although the direction of association was generally consistent, the magnitude of the effects should be interpreted in context.

### Supportive clinical and educational environments and professional identity

Clinical learning environment (*r* = 0.53, 95% CI: 0.18–0.77), social support (*r* = 0.52, 95% CI: 0.34–0.66), perceived hospital caring climate (*r* = 0.45, 95% CI: 0.31–0.57) and perceived teacher caring (*r* = 0.36, 95% CI: 0.22–0.50) were positively associated with professional identity. These findings suggest that professional identity is not solely an individual psychological process, but is also closely related to educational and organisational contexts.

Clinical internship is a key period during which nursing interns transition from the student role to the professional nursing role. A clinical environment that provides adequate learning opportunities, clear role guidance, positive interpersonal interactions and continuous support may be closely related to interns’ professional belonging and role internalisation. Social support may further reflect the combined influence of peers, schools, families and clinical mentors.

The positive associations of perceived teacher caring and perceived hospital caring climate with professional identity suggest that experiences of respect, understanding and recognition during clinical internship may be important external conditions for professional identity development. Nursing schools and hospitals may support this process by improving clinical learning environments, enhancing mentoring quality, establishing supportive feedback mechanisms and creating a caring organisational climate. Contextual factors such as region, hospital level and internship model should be considered when interpreting these findings.

### Humanistic competence, professional meaning-related experiences and professional identity

Humanistic caring ability, medical narrative competence and perceived professional benefit showed relatively strong positive associations with professional identity, with correlation coefficients of 0.77 (95% CI: 0.48–0.91), 0.64 (95% CI: 0.60–0.69) and 0.62 (95% CI: 0.55–0.66), respectively. Although conceptually distinct, these factors are all related to nursing interns’ understanding of professional value and meaning.

Humanistic caring ability showed the strongest association, suggesting that recognition and practice of the core values of nursing may be closely related to professional identity. Nursing is not limited to technical performance, but is a patient-centred professional practice that emphasises empathy, understanding and responsiveness. Interns with stronger humanistic caring ability may be more likely to experience the value of nursing through clinical care and nurse–patient interactions [25, 60].

The positive association between medical narrative competence and professional identity further suggests that understanding patients’ illness experiences, listening to their stories and engaging in reflection may deepen interns’ understanding of nursing value [12, 37]. Similarly, perceived professional benefit may enable interns to experience growth, achievement, recognition and professional value during internship, thereby supporting more stable identification with nursing [18, 36].

These findings indicate that professional identity development should not be limited to professional education or skills training. Humanistic education, narrative reflection, empathy training and positive professional experience may be integrated into internship training. However, the analysis of humanistic caring ability showed very high heterogeneity (I² = 99%), and its pooled estimate should therefore be interpreted in context. In contrast, findings for medical narrative competence (I² = 0%) and perceived professional benefit (I² = 8%) were more consistent.

### Role stress, anxiety and professional identity

Role stress (*r* = -0.32, 95% CI: -0.38 to -0.25) and anxiety (*r* = -0.14, 95% CI: -0.19 to -0.10) were negatively associated with professional identity. The association was stronger for role stress than for anxiety, suggesting that role-related stress may be an important negative experience during clinical internship.

Nursing interns are in a critical transition from the student role to the clinical nursing role. During this period, they must adapt to clinical systems, teamwork and work rhythms while facing increasing technical and professional responsibilities. Unclear role expectations, heavy task demands or insufficient clinical support may lead to role ambiguity, role conflict and role overload, which may be associated with lower professional belonging and professional identity [61, 53].

Although the negative association between anxiety and professional identity was relatively weak, it still suggests that negative emotional states may be related to lower professional identity [26, 55]. Anxiety may increase attention to work-related risks, personal errors and external evaluation, thereby intensifying perceived clinical stress. Role stress and anxiety may also interact with insufficient self-efficacy, inadequate support and difficulties in clinical adaptation.

Accordingly, support for professional identity development should include both the cultivation of positive resources and the identification and management of stress and negative emotions. Nursing schools and hospitals may reduce role stress and anxiety by clarifying internship role expectations, arranging task difficulty appropriately, strengthening mentoring support and providing psychological counselling. The low heterogeneity observed for role stress and anxiety suggests relatively consistent negative associations with professional identity.

### Non-significant finding

The association between clinical practice behaviour and professional identity was not statistically significant (*r* = 0.41, 95% CI: -0.03–0.73, *P* = 0.07), indicating that current evidence is insufficient to confirm a stable association between these two variables.

This non-significant result may be related to the complexity of the relationship between clinical practice behaviour and professional identity. The association may be influenced by clinical mentoring models, opportunities for skills training, baseline knowledge, individual initiative, communication competence and hospital support. In addition, the small number of included studies and high heterogeneity (I² = 96%) may have reduced the stability and precision of the pooled estimate.

Further studies are needed to clarify the relationship between clinical practice behaviour and professional identity and to examine the roles of clinical teaching models, internship stages and hospital support conditions.

### Demographic characteristics and internship-related background factors not included in the quantitative synthesis

Demographic characteristics and internship-related background factors that were not included in the quantitative synthesis were summarised descriptively. Overall, associations between general demographic characteristics and professional identity among nursing interns were inconsistent. In contrast, factors related to professional choice, future career intention, family occupational background and learning experience were more frequently reported to differ by professional identity level.

These findings suggest that professional identity may be more closely related to professional choice and socialisation experiences than to static demographic characteristics, such as sex, educational level or family residence. Nursing interns who voluntarily chose nursing, selected nursing as their first-choice major, had clear employment intentions or held positive perceptions of nursing career prospects may have entered clinical internship with clearer professional understanding and career expectations. In addition, family medical or nursing background, higher parental educational level, better academic performance, student cadre experience, participation in student associations, part-time work or social practice may provide greater access to occupational information, social interaction and self-management opportunities, which may support understanding of nursing roles and adaptation to clinical learning.From a nursing education perspective, professional identity support should not rely solely on demographic risk identification. Greater attention should be given to modifiable background factors, including professional choice guidance, career planning education, employment intention guidance, perceptions of career prospects and social practice opportunities. As the current evidence was mainly derived from group comparisons or regression analyses in individual studies, and variable definitions and grouping methods varied, these findings should be interpreted cautiously and further examined using standardised measures and longitudinal designs.

### Strengths and limitations

This study systematically synthesised evidence on factors associated with professional identity among nursing interns in China. By using Pearson’s correlation coefficient as a common effect size, quantitatively comparable factors were pooled, while factors reported in only one study were summarised descriptively. This approach provides a broad overview of the evidence and may help nursing educators and clinical managers understand professional identity from multiple perspectives, including psychological resources, clinical educational environments, caring support and professional meaning-related experiences.

Several limitations should be noted. First, all included studies were cross-sectional, so the findings indicate associations rather than causal relationships. The use of Pearson’s correlation coefficient as the only effect size may also have excluded studies that reported regression coefficients, path coefficients or structural equation modelling results without Pearson correlations, limiting the comprehensiveness of the evidence. Second, professional identity was measured using different instruments across studies. Differences in theoretical foundations, dimensions, items and scoring methods may affect comparability and contribute to heterogeneity. Third, some meta-analyses were based on only two studies, and several analyses showed substantial heterogeneity, which limits the robustness and interpretability of the pooled estimates. Finally, all included studies were conducted in China and most were published in Chinese; therefore, the applicability of the findings to other cultural contexts and nursing education systems requires further validation.

## Conclusion

This study systematically synthesised evidence on factors associated with professional identity among nursing interns in China. Professional identity was associated with multiple domains, including psychological resources, professional competence, clinical educational environments, caring support, professional meaning-related experiences and negative psychological states. These findings indicate that professional identity reflects not only interns’ recognition of nursing professional values, but also their clinical experiences, perceived support, understanding of nursing meaning and ability to cope with role transition and clinical stress.

Professional identity development should be integrated throughout nursing education and clinical internship. Nursing colleges and clinical internship institutions may support this process by providing structured feedback, progressive clinical tasks, resilience and coping skills training, optimising clinical learning environments, improving mentoring quality, and establishing continuous support mechanisms. Humanistic caring education, medical narrative training, reflective learning and positive professional experiences may also be incorporated into internship training. In addition, early identification and management of negative experiences, such as role stress, anxiety, transition shock, alienation and workplace bullying, should be emphasised.Supporting professional identity development may facilitate nursing interns’ transition from student to nurse, strengthen their sense of professional value, career confidence and future retention intention, and contribute to nursing workforce stability. Given that the included studies were mainly cross-sectional and that some factors were examined in a limited number of studies, further multicentre, large-sample and longitudinal studies are needed to verify these associations and clarify the underlying mechanisms.

## Supplementary Information

Additional File 1: Supplementary Material 1. Detailed search strategies.

Additional File 2: Supplementary Materials 2. MOOSE Checklist.

Additional File 3: Supplementary Materials 3. Demographic Background Professional Identity.

Additional file 4: Supplementary Materials 4. Quality assessment of the included studies.

## Data Availability

The datasets generated and/or analyzed during the current study are available from the Corresponding Author upon reasonable request.

## Abbreviations

PROSPERO: Prospective Register of Systematic Reviews
MOOSE: Meta-analyses Of Observational Studies in Epidemiology
AHRQ: Agency for Healthcare Research and Quality
PIQNS: Professional Identity Questionnaire for Nurse Students
NIS: Nurse Professional Identity Scale(developed by Liu Ling)
NIS-R: Revised Nurse Professional Identity Scale (developed and revised by Liu Shihui et al)
NIS-B: Professional Identity Questionnaire developed by Brown (Chinese version)
