# Supplementary material for: Factors associated with professional identity of nursing interns in China: a systematic review and meta-analysis
Source: BMC Med Educ. 2026 Jun 19;26:1394. doi: 10.1186/s12909-026-09692-9 (PMC13520297; doi:10.1186/s12909-026-09692-9)
Supplement: Supplementary file 2 [file 12909_2026_9692_MOESM1_ESM.docx]

**Supplementary Table S1. Detailed search strategies**

| Database | Search strategy | Records retrieved |
| --- | --- | --- |
| CNKI | 主题=(护理学生 OR 护理实习生 OR 实习护生) AND 主题=(职业认同 OR 专业认同) | 407 |
| PubMed | #1 ("nursing intern"[Title/Abstract] OR "nursing student"[Title/Abstract] OR "intern nurse"[Title/Abstract] OR "student nurse"[Title/Abstract])  #2 ("professional identity"[Title/Abstract] OR "professional recognition"[Title/Abstract] OR "professional confidence"[Title/Abstract] OR "professional commitment"[Title/Abstract])  #3 #1 AND #2 | 96 |
| wanfang | 主题=(护理学生 OR 护理实习生 OR 实习护生) AND 主题=(职业认同 OR 专业认同) | 1947 |
| VIP | #1 题名或关键词：护理学生 OR 护理实习生 OR 实习护生  #2 题名或关键词：职业认同 OR 专业认同  #3 #1 AND #2 | 183 |
| Cochrane Library | #1 (nursing intern OR nursing student OR intern nurse OR student nurse):ti,ab,kw  #2 (professional identity OR professional recognition OR professional confidence OR professional commitment):ti,ab,kw  #3 #1 AND #2 | 316 |
| CINAHL | #1 TX (nursing intern OR nursing student OR intern nurse OR student nurse)  #2 TX (professional identity OR professional recognition OR professional confidence OR professional commitment)  #3 #1 AND #2 | 563 |
| Embase | #1 ('nursing intern':ti,ab,kw OR 'nursing student':ti,ab,kw OR 'intern nurse':ti,ab,kw OR 'student nurse':ti,ab,kw)  #2 ('professional identity':ti,ab,kw OR 'professional recognition':ti,ab,kw OR 'professional confidence':ti,ab,kw OR 'professional commitment':ti,ab,kw)  #3 #1 AND #2 | 469 |
| Web of Science | #1 TS=(nursing intern OR nursing student OR intern nurse OR student nurse)  #2 TS=(professional identity OR professional recognition OR professional confidence OR professional commitment)  #3 #1 AND #2 | 440 |
| SinoMed | #1 "护理学生"[标题:智能] OR "护理实习生"[标题:智能] OR "实习护生"[标题:智能]  #2 "职业认同"[标题:智能] OR "专业认同"[标题:智能]  #3 #1 AND #2 | 145 |
