# Supplementary material for: Factors associated with professional identity of nursing interns in China: a systematic review and meta-analysis
Source: BMC Med Educ. 2026 Jun 19;26:1394. doi: 10.1186/s12909-026-09692-9 (PMC13520297; doi:10.1186/s12909-026-09692-9)
Supplement: Supplementary file 3 [file 12909_2026_9692_MOESM4_ESM.docx]

**Supplementary Table. Methodological quality assessment of included studies using the AHRQ checklist**

**Notes:** Y = Yes (1 point); N = No (0 points); U = Unclear (0 points); - = not applicable. Q11 was marked as not applicable for cross-sectional studies and was not counted. Quality levels were classified as high (8-11), moderate (4-7), and low (0-3). All quality levels were rechecked against the total scores and were consistent with the classification rule.

**Verification summary:** High-quality studies: 6; moderate-quality studies: 41; low-quality studies: 1; total studies: 48.

**AHRQ assessment items**

| **Item** | **Assessment item / scoring basis** |
| --- | --- |
| Q1 | Define the source of information. |
| Q2 | List inclusion/exclusion criteria or refer to previous publications. |
| Q3 | Indicate time period used for identifying participants. |
| Q4 | Indicate whether participants were consecutive if not population-based. |
| Q5 | Indicate whether evaluators of subjective components were masked. |
| Q6 | Describe quality assurance assessments. |
| Q7 | Explain participant exclusions from analysis. |
| Q8 | Describe how confounding was assessed and/or controlled. |
| Q9 | Explain how missing data were handled, if applicable. |
| Q10 | Summarize response rates and data completeness. |
| Q11 | Clarify incomplete data/follow-up if applicable; marked as not applicable for cross-sectional studies. |

**AHRQ scoring matrix**

| **First author / year** | **Q1** | **Q2** | **Q3** | **Q4** | **Q5** | **Q6** | **Q7** | **Q8** | **Q9** | **Q10** | **Q11** | **Total** | **Level** |
| --- | --- | --- | --- | --- | --- | --- | --- | --- | --- | --- | --- | --- | --- |
| Li 2024 | Y | Y | Y | Y | U | N | Y | N | Y | Y | - | 7 | Moderate |
| Sun 2016 | Y | U | Y | Y | U | N | N | N | N | Y | - | 4 | Moderate |
| Liu 2020 | Y | N | Y | Y | U | N | N | Y | N | N | - | 4 | Moderate |
| Hu 2025 | Y | Y | Y | Y | U | N | Y | N | N | N | - | 5 | Moderate |
| Fu 2019 | Y | Y | Y | Y | U | N | Y | Y | N | Y | - | 7 | Moderate |
| Lei 2024 | Y | Y | Y | Y | U | N | Y | N | N | Y | - | 6 | Moderate |
| Li 2019 | Y | Y | Y | Y | U | N | Y | N | N | Y | - | 6 | Moderate |
| Liu 2023 | Y | Y | Y | Y | U | N | Y | Y | N | Y | - | 7 | Moderate |
| Luo 2025 | Y | Y | Y | Y | U | N | Y | N | N | Y | - | 6 | Moderate |
| Ou 2020 | Y | Y | Y | Y | U | N | Y | N | N | Y | - | 6 | Moderate |
| Nong 2022 | Y | Y | Y | Y | U | N | Y | N | N | Y | - | 6 | Moderate |
| Peng 2022 | Y | Y | Y | Y | U | N | Y | Y | N | Y | - | 7 | Moderate |
| Wang 2022 | Y | Y | Y | Y | U | N | Y | Y | N | Y | - | 7 | Moderate |
| Wang 2024 | Y | Y | Y | Y | U | N | Y | N | N | Y | - | 6 | Moderate |
| Xu 2023 | Y | Y | Y | Y | U | N | Y | N | N | Y | - | 6 | Moderate |
| Xu 2023 | Y | Y | Y | Y | U | N | Y | Y | N | Y | - | 7 | Moderate |
| Yu 2024 | Y | U | Y | Y | U | N | Y | Y | N | Y | - | 6 | Moderate |
| Yuan 2021 | Y | U | Y | Y | U | N | Y | Y | N | Y | - | 6 | Moderate |
| Zhang 2022 | Y | Y | Y | Y | U | N | Y | Y | N | Y | - | 7 | Moderate |
| Zhang 2024 | Y | Y | Y | Y | U | N | N | Y | N | N | - | 5 | Moderate |
| Zhao 2022 | Y | Y | Y | Y | U | N | Y | Y | Y | Y | - | 8 | High |
| Dou 2018 | Y | Y | Y | Y | U | N | Y | N | N | Y | - | 6 | Moderate |
| Guo 2017 | Y | U | Y | Y | U | N | N | N | N | Y | - | 4 | Moderate |
| He 2021 | Y | N | Y | Y | U | Y | N | Y | Y | N | - | 6 | Moderate |

| **First author / year** | **Q1** | **Q2** | **Q3** | **Q4** | **Q5** | **Q6** | **Q7** | **Q8** | **Q9** | **Q10** | **Q11** | **Total** | **Level** |
| --- | --- | --- | --- | --- | --- | --- | --- | --- | --- | --- | --- | --- | --- |
| He 2022 | Y | Y | Y | Y | U | N | Y | Y | Y | Y | - | 8 | High |
| Liu 2015 | Y | Y | Y | Y | U | N | N | N | N | Y | - | 5 | Moderate |
| Meng 2024 | Y | Y | Y | Y | U | N | Y | Y | N | Y | - | 7 | Moderate |
| Shi 2021 | Y | Y | Y | Y | U | N | Y | Y | N | Y | - | 7 | Moderate |
| Su 2024 | Y | Y | Y | Y | U | N | Y | Y | N | Y | - | 7 | Moderate |
| Sun 2020 | Y | Y | Y | Y | U | Y | N | N | Y | Y | - | 7 | Moderate |
| Sun 2023 | Y | Y | Y | Y | U | Y | Y | Y | Y | Y | - | 9 | High |
| Wang 2021 | Y | N | N | Y | U | N | N | N | N | Y | - | 3 | Low |
| Wang 2018 | Y | U | N | Y | U | Y | N | Y | N | Y | - | 5 | Moderate |
| Wang 2023 | Y | Y | Y | Y | U | Y | Y | N | Y | Y | - | 8 | High |
| Xiao 2016 | Y | Y | Y | Y | U | Y | N | N | Y | Y | - | 7 | Moderate |
| Xu 2019 | Y | N | Y | Y | U | Y | N | Y | N | Y | - | 6 | Moderate |
| Yu 2017 | Y | U | Y | Y | U | N | N | N | N | Y | - | 4 | Moderate |
| Zhang 2016 | Y | U | Y | Y | U | Y | Y | Y | N | Y | - | 7 | Moderate |
| Lin 2025 | Y | Y | Y | Y | U | N | Y | Y | N | Y | - | 7 | Moderate |
| Zhang 2025 | Y | Y | Y | Y | U | Y | Y | Y | Y | Y | - | 9 | High |
| Sun 2016 | Y | U | Y | Y | U | Y | N | Y | N | Y | - | 6 | Moderate |
| Yang 2025 | Y | Y | Y | Y | U | N | N | N | Y | N | - | 5 | Moderate |
| Zhu 2024 | Y | Y | Y | Y | U | N | Y | N | N | Y | - | 6 | Moderate |
| Yao 2021 | Y | Y | Y | Y | U | Y | Y | N | Y | Y | - | 8 | High |
| Lin 2023 | Y | Y | Y | Y | U | Y | N | Y | N | Y | - | 7 | Moderate |
| Jiang 2025 | Y | Y | Y | Y | U | N | Y | Y | N | Y | - | 7 | Moderate |
| Wang 2025 | Y | Y | Y | Y | U | N | Y | N | Y | Y | - | 7 | Moderate |
| Li 2022 | Y | N | Y | Y | U | N | N | Y | N | Y | - | 5 | Moderate |

**Evaluation basis**

*The following brief notes explain the main reasons for the item-level judgements and overall ratings.*

| **First author / year** | **Total** | **Level** | **Evaluation basis** |
| --- | --- | --- | --- |
| Jiang 2025 | 7 | Moderate | Clear data source, timing, tools, eligibility and response rate; no blinding/quality assurance or missing-data handling reported. |
| Wang 2025 | 7 | Moderate | Clear sampling, timing, eligibility, ethics, tools, quality checks for online responses and response rate; no confounder control reported. |
| Li 2022 | 5 | Moderate | Multi-region sample, timing, tools and regression reported; eligibility, exclusion reasons and  missing-data handling were insufficient. |
| Li 2024 | 7 | Moderate | Clear eligibility, sample size estimation, tools, online response checks and response rate; no confounder control reported. |
| Sun 2016 | 4 | Moderate | Basic data source, timing, tools and response rate reported; eligibility was incomplete and no  confounder control or missing-data handling was described. |
| Liu 2020 | 4 | Moderate | Data source, timing, tools and regression were  reported; eligibility criteria, response rate,  exclusion reasons and missing-data handling were not clearly reported. |
| Hu 2025 | 5 | Moderate | Eligibility, sample size and instruments were reported; no response rate, confounder control, quality assurance or missing-data handling was reported. |
| Fu 2019 | 7 | Moderate | Sampling, eligibility, sample size, validated tools, response rate and regression with demographic  control were reported; missing-data handling was unclear. |
| Lei 2024 | 6 | Moderate | Eligibility, timing, tools and response rate were reported; no confounder control, limited missing- data handling and limited measurement quality assurance. |
| Li 2019 | 6 | Moderate | Eligibility, timing, tools, data collection and  response rate were reported; no clear confounder control or missing-data handling. |

| **First author / year** | **Total** | **Level** | **Evaluation basis** |
| --- | --- | --- | --- |
| Liu 2023 | 7 | Moderate | Eligibility, multi-hospital sampling, tools, ethics, response rate and regression were reported; no blinding, quality assurance or missing-data  handling reported. |
| Luo 2025 | 6 | Moderate | Sampling, timing, eligibility, tools and response rate were reported; no confounder control or  missing-data handling. |
| Ou 2020 | 6 | Moderate | Eligibility, data collection, tools and response rate were reported; no blinding, quality assurance,  confounder control or missing-data handling. |
| Nong 2022 | 6 | Moderate | Eligibility, timing, tools and response rate were  reported; no confounder control, quality assurance or missing-data handling. |
| Peng 2022 | 7 | Moderate | Eligibility, timing, response checks, response rate and hierarchical regression were reported; no  blinding or detailed missing-data handling. |
| Wang 2022 | 7 | Moderate | Sampling, timing, eligibility, validated tools, response rate and multiple regression were  reported; no quality assurance or missing-data handling. |
| Wang 2024 | 6 | Moderate | Eligibility, exclusion criteria, sample size, tools and response rate were reported; no confounder control or missing-data handling. |
| Xu 2023 | 6 | Moderate | Eligibility, timing, instruments and response rate were reported; no confounder control, quality  assurance or missing-data handling. |
| Xu 2023 | 7 | Moderate | Eligibility, tools, response checks, response rate and multiple regression were reported; no quality assurance or missing-data handling. |
| Yu 2024 | 6 | Moderate | Timing, tools, response checks, response rate and regression with general data were reported;  eligibility criteria were incomplete and missing- data handling unclear. |
| Yuan 2021 | 6 | Moderate | Data source, instruments, response rate and  regression were reported; eligibility criteria and missing-data handling were not fully clear. |

| **First author / year** | **Total** | **Level** | **Evaluation basis** |
| --- | --- | --- | --- |
| Zhang 2022 | 7 | Moderate | Eligibility, tools, response rate and hierarchical  regression with general variables were reported; no quality assurance or missing-data handling. |
| Zhang 2024 | 5 | Moderate | Eligibility, tools and multiple regression were reported; response rate, exclusion reasons and missing-data handling were unclear. |
| Zhao 2022 | 8 | High | Eligibility, sample size estimation, tools, response rate, required-response design and multiple  regression were reported; only blinding and extra quality assurance were not reported. |
| Dou 2018 | 6 | Moderate | Eligibility, tools, on-site survey and response rate were reported; no confounder control, quality  assurance or missing-data handling. |
| Guo 2017 | 4 | Moderate | Sampling, timing, tools and response rate were  reported; eligibility was incomplete and no  exclusion reasons, confounder control or missing- data handling were reported. |
| He 2021 | 6 | Moderate | Timing, tools, field checking and regression with demographic control were reported; eligibility, response rate and exclusion reasons were not  reported. |
| He 2022 | 8 | High | Stratified sampling, eligibility, tools, response rate, exclusion of invalid questionnaires and regression were reported; no blinding or extra quality  assurance. |
| Liu 2015 | 5 | Moderate | Eligibility, tools and response rate were reported; no exclusion reasons, confounder control, quality assurance or missing-data handling. |
| Meng 2024 | 7 | Moderate | Random sampling, timing, eligibility, tools,  response count and regression were reported; no quality assurance or missing-data handling. |
| Shi 2021 | 7 | Moderate | Sample size estimation, eligibility, tools, response rate and multiple regression were reported; no  quality assurance or missing-data handling. |
| Su 2024 | 7 | Moderate | Eligibility, sample size estimation, online response control, response rate and regression were  reported; no quality assurance or missing-data handling. |

| **First author / year** | **Total** | **Level** | **Evaluation basis** |
| --- | --- | --- | --- |
| Sun 2020 | 7 | Moderate | Eligibility, tools, on-site quality checking,  completion of missing items and response rate were reported; no confounder control and  exclusion reasons unclear. |
| Sun 2023 | 9 | High | Eligibility, ethics, timing, validated tools, detailed quality control, exclusion rules, response rate,  double data checking and regression were reported. |
| Wang 2021 | 3 | Low | Basic source, tools and response rate were  reported, but eligibility, timing, quality control, exclusion reasons, confounder control and  missing-data handling were not reported. |
| Wang 2018 | 5 | Moderate | Tools, trained data collection, double entry,  response rate and regression with demographic control were reported; eligibility, timing and exclusion reasons were incomplete. |
| Wang 2023 | 8 | High | Eligibility, sample size, ethics, validated tools, trained investigators, double entry and invalid questionnaire handling were reported; no  confounder control. |
| Xiao 2016 | 7 | Moderate | Eligibility, timing, tools, trained investigators,  checking for missing items and response rate were reported; no confounder control and exclusion  reasons unclear. |
| Xu 2019 | 6 | Moderate | Timing, tools, double checking/data entry, response rate and multiple regression were reported; eligibility, exclusion reasons and missing-data handling were unclear. |
| Yu 2017 | 4 | Moderate | Data source, timing, tools and response rate were reported; eligibility incomplete and no quality  assurance, exclusion reasons, confounder control or missing-data handling. |
| Zhang 2016 | 7 | Moderate | Timing, sample size, inclusion conditions, tools, on-site collection, exclusion of incomplete  questionnaires, double entry and regression were reported; no complete exclusion criteria or  missing-data handling. |

| **First author / year** | **Total** | **Level** | **Evaluation basis** |
| --- | --- | --- | --- |
| Lin 2025 | 7 | Moderate | Cross-sectional design, timing, eligibility, sample size, tools, response rate and adjusted  mediation/regression analyses were reported;  quality assurance and missing-data handling were unclear. |
| Zhang 2025 | 9 | High | Eligibility, sample size, online quality control,  pilot testing, exclusion reasons, response rate,  prevention of incomplete submissions and adjusted analyses were reported. |
| Sun 2016 | 6 | Moderate | Timing, tools, supervised data collection,  ethics/consent and regression were reported; eligibility and missing/exclusion details were incomplete. |
| Yang 2025 | 5 | Moderate | Design, timing, eligibility, sample size, ethics,  tools and missing-data strategy were reported;  response rate, exclusion reasons, quality assurance and adjusted analysis were limited. |
| Zhu 2024 | 6 | Moderate | Design, timing, eligibility, sample size, tools,  response rate and exclusion of low-quality  responses were reported; no confounder control or missing-data handling. |
| Yao 2021 | 8 | High | Eligibility, ethics, team roles, electronic  completeness check, invalid questionnaire  exclusion, large sample and SEM were reported; no confounder control. |
| Lin 2023 | 7 | Moderate | Eligibility, sample size, feasibility check, tools, response rate and mediation model with sex  covariate were reported; exclusion reasons and missing-data handling were limited. |
